# Persistent household food insecurity, HIV, and maternal stress in Peri-Urban Ghana

**DOI:** 10.1186/1471-2458-13-215

**Published:** 2013-03-11

**Authors:** Jonathan Garcia, Amber Hromi-Fiedler, Robert E Mazur, Grace Marquis, Daniel Sellen, Anna Lartey, Rafael Pérez-Escamilla

**Affiliations:** 1 Center for Interdisciplinary Research on AIDS, Yale School of Public Health, New Haven, CT, USA; 2 Yale School of Public Health, 135 College Street, Suite 200, New Haven, CT, 06510, USA; 3 Iowa State University, Ames, IA, USA; 4 McGill University, Montreal, QC, Canada; 5 University of Toronto, Toronto, ON, Canada; 6 University of Ghana-Legon, Legon, Ghana

**Keywords:** Maternal stress, Food Insecurity, HIV/AIDS, Ghana, Syndemic

## Abstract

**Background:**

The mental health of caregivers has been shown to be important for improving HIV prevention and treatment. Household food insecurity affects hundreds of millions of individuals in Sub-Saharan Africa, a region that experiences a disproportionate burden of the HIV pandemic. Both maternal HIV diagnosis and household food insecurity may be linked with maternal stress. This in turn may lead to unhealthy coping behaviors. We examined the independent associations of HIV, persistent household food insecurity and the synergistic effect of both on maternal stress.

**Methods:**

Ghanaian women recruited prenatally from hospitals offering voluntary counseling and testing (VCT) were followed for 12 months after childbirth (N = 232). A locally adapted 7-item version of the US Household Food Security Survey Module was applied at four time points postpartum. We dichotomized participant households as being persistently food insecure (i.e., food insecure at each time point) or not (i.e., food secure at any time point). We dichotomized participant women as not perceiving vs. perceiving stress at 12 months postpartum in reference to the median sample score on the 4-item Cohen’s stress scale. Binary multivariate logistic regression models were used to assess the independent and interactive effects of maternal HIV and persistent household food insecurity on maternal stress.

**Results:**

The proportion of HIV-positive women that lived in severe food insecure households increased over time. By contrast, the HIV-negative group living in severely food insecure households experienced a steady decline across time. HIV-infection (AOR = 2.31, 95% CI 1.29-4.12) and persistent household food insecurity (AOR = 3.55, 95% CI 1.13-11.13) were independently associated with maternal stress in a multivariate model. Being both HIV-positive and persistently food insecure strongly and synergistically increased the risk for maternal perceived stress (AOR = 15.35, 95% CI 1.90-124.14).

**Conclusion:**

In agreement with syndemic theory there is a powerful synergism between maternal HIV diagnosis and household food insecurity on maternal stress. Comprehensive multi-dimensional intervention studies are needed to better understand how to reduce stress among HIV-positive women living in persistently food insecure households and how to reduce the likelihood of food insecurity in HIV-affected households in Sub-Saharan Africa.

## Background

Household food insecurity (HFI) is defined as the limited or uncertain availability of foods that are nutritionally adequate, safe, and acquired through socially acceptable ways [1]. Globally there is an overlap between HFI and HIV/AIDS [2,3]. Both HFI and HIV/AIDS have been identified not only as major independent biological but also as mental health stressors [4,5]. There are reasons to expect for the joint effect of these two conditions to become synergistic when simultaneously present. HIV/AIDS and food insecurity in Sub Saharan Africa have been characterized as a syndemic not only because they coexist but also they are both rooted in social inequalities and, as in a vicious cycle, the presence of one condition is likely to exacerbate the second condition [6-8]. On one hand HIV can have a profound effect on the structure and functioning of households [8]. For example, a woman diagnosed with HIV may be abandoned by her husband and experience a significant reduction in social support [8]. HIV may also weaken her immune system and ability to work productively [8] and follow sustainable livelihood strategies conducive to good health outcomes [7]. As a result maternal HIV is likely to worsen the economic situation of a household and to negatively affect its access to food [6,7]. On the other hand women living in food insecure households may engage in risky economic generation activities (e.g., unsafe sexual practices) that may increase their risk of acquiring HIV [6,9]. In Sub-Saharan Africa poverty-related migration might represent a coping mechanism that has a negative effect on well-being of women and children [10]. For instance, male labor migration has been linked to maternal HIV and household food insecurity status in Sub-Saharan Africa, especially in places where there is a high level of gender inequality [7]. And among women who are already infected with HIV food insecurity may prevent them from taking adequate care of HIV and as result transition more rapidly into full blown AIDS. Food insecurity may foster the further infection of other individuals by HIV positive women who follow risky sexual behaviors as a result of their lack of access to food. In sum, both maternal HIV and food insecurity are major household stressors that can be expected to affect maternal mental health [7]. Because both conditions exacerbate each other we hypothesize that they may act synergistically on affecting maternal stress. Answering this innovative question is of considerable public health significance as poverty related maternal stress has been linked with other maternal mental health problems (e.g., depression) and poor child development [11].

The primary objective of this paper is to examine the independent and synergistic associations between HIV status, persistent household food insecurity (PHFI), and perceived stress in Ghanaian mothers of young children. We hypothesize that HIV and food insecurity might create a synergy on maternal stress that is greater than the additive influence of these risk factors. Even though Ghana is a “low” HIV prevalence country the district where the study took place had significant HIV rates at the time of the study. The prevalence of HIV in the Ghanaian district of Manya Krobo, where this study took place, was 8.9% in 2007 among pregnant women [12]. This prevalence was indeed much higher than the prevalence among pregnant women nationally, which was estimated to be 1.8% in 2008 [13].

## Methods

### Procedures

The “Research to Improve Infant Nutrition and Growth” (RIING) study was carried out between 2004 and 2009. This study was approved by the ethics boards of The University of Ghana-Legon, Iowa State University, The University of Connecticut, and McGill University. The research team enrolled women who were receiving prenatal care visits in the Eastern Region in Ghana [14]. Women were recruited from three public hospitals that provided voluntary counseling and testing (VCT) and offered antiretroviral therapy for the prevention of mother-to-child transmission. However this consisted only of nevirapine administered to the mother at the time of labour and a single dose administered to the infant at birth. The women went through the regular Ghana Health Service (GHS) antenatal clinic procedures which included voluntary pretest counselling to offer HIV testing. Before 2005, the opt-in voluntary counselling and testing (VCT) policy was in place, which required offering of information on voluntary counselling and women had to agree to be tested. Testing acceptance rate for one of the recruiting hospitals was 3.2% in 2004. Due to the low acceptance rate, after 2005 the GHS introduced the opt–out policy for which women were tested for HIV unless they requested not to be. This new policy resulted in a substantial increase in the proportion of pregnant women being tested for HIV (acceptance rate increased to 19.5% in 2006 for the same hospital).

Recruitment into the study was done in partnership with the hospital nursing staff responsible for VCT in the three participating hospitals. Participants were followed at the time of birth, and subsequently every three months for a 12-month period. All women over the age of 18 who agreed to be tested for HIV as well as those who refused to be tested were included in the study (N = 547). For the purpose of these analyses, only the women who accepted to be tested (positive or negative) and who answered the food security module at any time point postpartum were included (N = 285). Because of missing data (18.6%), a total number of 232 participants out of the 285 of the initial analytical sample were analyzed.

### Measures

#### Persistent Household Food Insecurity (PHFI)

Household food security was measured with an adapted 7-item scale derived from the 18-item USDA Household Food Security Survey Module (HFSSM) [15]. This experience-based scale was selected because of the expertise of the senior author (RPE) adapting the HFSSM in low- and middle- income countries [16]. Rasch modeling [17,18] was applied to understand the psychometric behavior of the 8 adult items included in the RIING Household Food Insecurity Measurement Scale (RHFIS) across different time points. Except for item # 3 (tapping the construct of ‘balanced’ meals), the rest of the 7 items had adequate psychometric behavior across time and their severity scores loaded consistent with theoretical expectations (see Appendix for specific questions). Scale item Rasch maps suggested the following algorithm to further classify households according to their food insecurity level: secure (0); mild/moderate FI (1–4); severe FI (5–7) [Pérez-Escamilla R: *Psychometric properties of the RIING Household Food Insecurity Measurement Scale: Adult items,*unpublished].

The household food insecurity independent variable was dichotomous classifying households as either PHFI or not. PHFI was defined as households that were food insecure (being mildly/moderately or severely food insecure) at all time points post-partum (PP) (i.e., months 3, 6, 9, and 12 pp). Only households that had data for *all four* of these time points were included in the *persistent* household food insecurity (PHFI) analyses (n = 232).

#### Stress scale

Perceived stress was assessed using the Cohen, Kamarck, and Mermenlstein [19] 4-item Perceived Stress Scale. Respondents were asked if they (a) were effectively coping with important changes in life, (b) had confidence about their own ability to handle personal problems, (c) had the inability to control the important things in life, (d) had an inability to overcome difficulties. A 5-point scale response option was used for each question (1 = Never, 2 = Only once or twice, 3 = At least once a week, 4 = More than once a week, 5 = Almost daily) in reference to the last month. The responses for items 1 and 2 were reversed, and a summative score was created to compute the stress level (score ranged from 4–20). The scale’s Cronbach’s Alpha was 0.91 indicating adequate internal validity. A dichotomous variable was created using the sample’s median score of 4, and women were classified as perceiving stress (a score greater than 4) or not perceiving stress.

#### Socioeconomic and demographic variables

Data analyses adjusted for five socioeconomic and demographic variables that were hypothesized to be potential confounders of the relationships between HIV status, PHFI, and maternal stress. The covariates selected were marital status, maternal age, education, having electricity in the home, and household size (computed by adding the number of people residing in the household where the respondent lived).

### Data analyses

Data analyses were conducted using SPSS for Windows 17.0. The outcome variable analyzed was maternal stress status at one year postpartum (pp). Bivariate analyses were conducted to compare: (a) HIV-positive mothers to HIV-negative mothers according to their demographic and socioeconomic background characteristics; (b) the background characteristics of women based on their stress status (stress vs. no stress); and (c) the percentages of women who lived in households experiencing severe household food insecurity by HIV-status from enrollment to 12-months pp. Four adjusted multivariate logistic regression models were run to test associations between HIV, PHFI and stress. The first model included both HIV status and PHFI as main independent variables. The second model excluded HIV status to examine whether HIV mediated the relationship between PHFI and stress. Likewise, the third model excluded PHFI to determine whether this variable mediated the relationship between HIV and stress. The fourth model tested for the synergistic effect of HIV and PHFI (i.e., influence of their simultaneous presence) on maternal stress. We initially considered including a four-level independent variable to represent the four different possible combinations of HIV (positive/negative) and PHFI (present/absent). However, because of the low prevalence of mothers who were both PHFI and HIV-negative (n = 7, 2.5% of sample) this variable was collapsed into three categories: a) HIV-positive and PHFI, b) HIV-positive and not PHFI or HIV-negative and PHFI, c) HIV-negative and not PHFI.

## Results

The average participant’s age was 28.4 (SD 5.8). Women had an average of 8.0 (SD 4.3) years of education. In this sample, 10.3% of women had no education, and 10.8% of the women experience PHFI. The average household size was 6.0 persons (SD 2.4). Twenty percent of households had 1–4 persons, 37.9% 5–6 persons, and 41.8% more than 7 persons. The great majority (81%) of women lived in households that had electricity. Twenty-six percent of women reported not having a spouse. In this sample, there were 38% HIV-positive women and 62% HIV-negative women. As expected HIV-positive women were more socio-economically vulnerable (Table 1). Likewise, women with stress were more likely to be HIV-positive, to live in larger households and to live in PHFI households (Table 2).

**Table 1:** Sample characteristics stratified by maternal HIV status

**Variable**	**Status**	**Combined N = 232**	**HIV-positive n = 89**	**HIV-negative n = 143**	***X***^***2 ***^***(p-value)***
**Maternal Stress**^**1**^	Yes	58.2%	69.7%	51.0%	7.81 (0.004)
	No	42.1%	30.3%	49.0%	
**Persistent Household Food Insecurity (PHFI)**	PHFI	10.8%	20.2%	4.9%	13.40 (0.000)
	Not PHFI	89.2%	79.8%	95.1%	
**Spouse**	Yes	73.7%	62.9%	80.4%	8.67 (0.003)
	No	26.3%	37.1%	19.6%	
**Maternal Education (y)**	0	10.3%	16.9%	6.3%	14.03 (0.003)
	1–6	23.7%	31.5%	18.9%	
	7–9	41.4%	32.6%	46.9%	
	10–12	24.6%	19.1%	28.0%	
**Electricity**	Yes	81.0%	74.2%	85.3%	4.44 (0.035)
	No	19.0%	25.8%	14.7%	
**Household Size**	1–4	20.3%	18.0%	21.7%	0.614 (0.736)
	5–6	37.9%	40.4%	36.4%	
	> 6	41.8%	41.6%	42.0%	
**Maternal Age (y)**	18–24	27.2%	28.1%	26.6%	4.60 (0.204)
	25–28	23.3%	27.0%	21.0%	
	29–32	22.8%	25.8%	21.0%	
	> 32	26.7%	19.1%	31.5%	

^1^Maternal stress measured with Cohen’s et al. [19] 4-item Perceived Stress Scale. A 5-point scale response option was used for each question (1 = Never, 2 = Only once or twice, 3 = At least once a week, 4 = More than once a week, 5 = Almost daily) in reference to the last month. The responses for items 1 and 2 were reversed, and a summative score was created to compute the stress level (score ranged from 4–20). Women were classified as experiencing stress if they had a score above 4.

**Table 2:** **Sample characteristics stratified by maternal stress level**^**1**^

**Variable**	**Status**	**Combined*(N = 232)***	**No Stress (%) (n = 97)**	**Stress (%) (n = 135)**	***X***^***2 ***^***(p-value)***
**Maternal HIV**	Positive	38.4%	27.8%	45.9%	7.81 (0.005)
	Negative	61.6%	72.2%	27.8%	
**Persistent Household Food Insecurity (PHFI)**	PHFI	10.8%	4.1%	15.6%	7.67 (0.006)
	Not PHFI	89.2%	95.9%	84.4%	
**Spouse**	Yes	73.7%	71.1%	77.3%	1.12 (0.289)
	No	26.3%	28.9%	22.7%	
**Maternal Education (y)**	0	9.8%	9.3%	11.1%	0.22 (0.975)
	1–6	24.0%	23.7%	25.2%	
	7–9	42.5%	42.3%	40.7%	
	10–12	23.6%	24.7%	24.4%	
**Electricity at home**	Yes	81.0%	84.5%	78.5%	1.33 (0.249)
	No	19.0%	15.5%	21.5%	
**Household Size**	1–4	20.9%	29.9%	13.3%	9.75 (0.008)
	5–6	37.9%	32.0%	42.2%	
	> 6	41.8%	38.1%	44.4%	
**Maternal Age (y)**	18–24	27.2%	28.9%	25.9%	1.97 (0.579)
	25–28	23.3%	25.8%	21.5%	
	29–32	22.8%	18.6%	25.9%	
	> 32	26.7%	26.8%	26.7%	

^1^Maternal stress measured with Cohen’s et al. [19] 4-item Perceived Stress Scale. A 5-point scale response option was used for each question (1 = Never, 2 = Only once or twice, 3 = At least once a week, 4 = More than once a week, 5 = Almost daily) in reference to the last month. The responses for items 1 and 2 were reversed, and a summative score was created to compute the stress level (score ranged from 4–20). Women were classified as experiencing stress if they had a score above 4.

**Figure 1:**
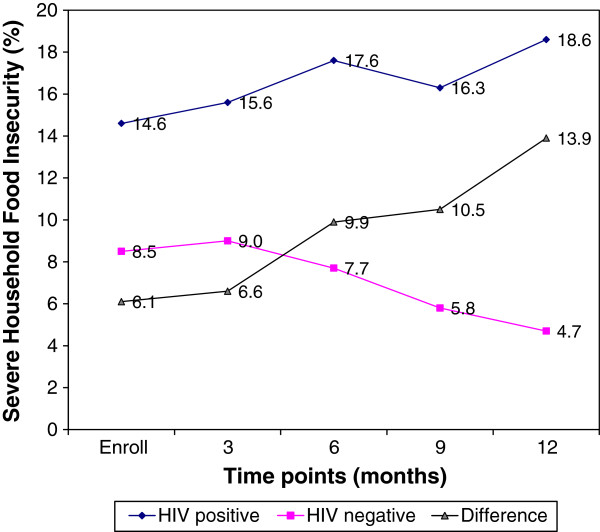
Prevalence of Severe Household Food Insecurity (PSHFI) from enrollment to 12 months post-enrollment as a function of maternal HIV status in peri-urban Ghana.

Figure 1 presents the pattern of severe household food insecurity by HIV status across time, i.e., from prenatal to 12 months pp. The percentage of HIV-positive women who lived in severely food insecure households increased over time. By contrast, the HIV-negative group experienced a steady decline across time in percentages living in severe food insecure households. The magnitude of the between-group difference in severe household food insecurity across time increased from 6.1% at enrollment to 13.9% at 12-months.

**Figure 2:**
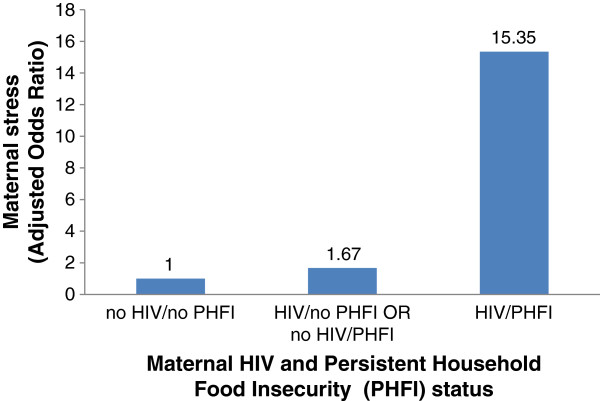
Risk of maternal perceived stress as a function of maternal HIV and Persistent Household Food Insecurity (PHFI) status in peri-urban Ghana.

Multivariate model 1 (Table 3) showed that the odds for experiencing maternal stress were twice as high among HIV-positive women compared with their HIV-negative counterparts. Women living in persistent food insecure households were 2.85 times more likely to experience stress compared to those who were not from persistently food insecure households. Model 2 (excluding HIV status) found that the odds of experiencing stress were 3.55 times higher among women living in persistently food insecure households compared to those who did not live in these households. Model 3 (excluding PHFI) found that the odds of experiencing stress were twice as high among HIV positive mothers than among HIV-negative mothers. Model 4 (testing the synergistic effect of HIV and PHFI) showed that the odds of experiencing stress were 15 times higher when both HIV and PHFI were simultaneously present (vs. when none of these stressors was present) (Figure 2).

**Table 3:** **Main effects and interaction of HIV status and persistent household food insecurity (PHFI) on Maternal Stress in Ghana’s Eastern Region**^**1**^**(N = 232)**

**Category**	**Status**	**Model 1 (N = 232)** **OR (95% CI)**	**Model 2 (N = 232)** **OR (95% CI)**	**Model 3 (N = 232)** **OR (95% CI)**	**Model 4 (N = 232)** **OR (95% CI)**
**HIV**	Positive	2.03 *** (1.09–3.77)	--	2.31 *** (1.29–4.12)	--
	Negative	Reference	--	Reference	--
**Persistent Household Food Insecurity (PHFI)**	PHFI	2.85** (0.89–9.14)	3.55 *** (1.13–11.13)	--	--
	Not PHFI	Reference	Reference	--	--
**HIV and PHFI**	HIV + and PHFI	--	--	--	15.35 *** (1.90–124.14)
	HIV + and not PHFI or HIV – and PHFI	--	--	--	1.67 (0.91–3.07)
	HIV – and not PHFI	--	--	--	1.00
**Maternal Age**	17–24	0.875 (0.39–1.98)	0.94 (0.42–2.10)	0.79 (0.36–1.73)	0.89 (0.40–2.01)
	25–28	0.92 `(0.41–2.08)	1.02 (0.46–2.29)	0.83 (0.38–1.82)	0.96 (0.42–2.15)
	29–32	1.31 (0.58–2.95)	1.38 (0.62–3.07)	1.33 (0.61–2.90)	1.34 (0.59–3.01)
	32 +	1.00	1.00	1.00	1.00
**Maternal Education**	None	0.71 (0.23–2.20)	0.88 (0.29–2.63)	0.75 (0.25–2.25)	0.68 (0.21–2.13)
	Primary (1–6 y)	0.77 (0.34–1.75)	0.90 (0.41–2.00)	0.77 (0.35–1.70)	0.82 (0.36–1.84)
	Junior Secondary (7–9 y)	1.06 (0.52–2.14)	1.07 (0.53–2.15)	1.03 (0.53–2.03)	1.08 (0.53–2.20)
	Senior Secondary (10-12y) +	1.00	1.00	1.00	1.00
**Household Size**	1–4	0.44 *** (0.20–0.97)	0.46 *** (0.21–0.99)	0.50** (0.24–1.03)	0.44 *** (0.20–0.96)
	5–6	1.21 (0.64–2.27)	1.23 (0.66–2.31)	1.23 (0.67–2.25)	1.20 (0.64–2.25)
	> 6	1.00	1.00	1.00	1.00
**Spouse**	No	1.10 (0.53–2.26)	1.23 (0.61–2.49)	1.24 (0.62–2.47)	1.07 (0.52–2.22)
	Yes	1.00	1.00	1.00	1.00
**Electricity at home**	No	1.20 (0.56–2.57)	1.22 (0.57–2.60)	1.39 (0.68–2.87)	1.21 (0.56–2.60)
	Yes	1.00	1.00	1.00	1.00

Multivariate binary logistic regression analyses.

^1^Maternal stress measured with Cohen’s et al. (19) 4-item Perceived Stress Scale. A 5-point scale response option was used for each question (1 = Never, 2 = Only once or twice, 3 = At least once a week, 4 = More than once a week, 5 = Almost daily) in reference to the last month. The responses for items 1 and 2 were reversed, and a summative score was created to compute the stress level (score ranged from 4–20). Women were classified as experiencing stress if they had a score above 4.

*** Significant at p < 0.05.

** Significant at p < 0.10.

## Discussion

To our knowledge, this is the first study to examine the independent and combined influence of both HIV and PHFI on maternal stress. Consistent with the syndemic and the ‘new variant famine’ frameworks (6–8), findings strongly suggest that PHFI and HIV amplify the influence of each other on maternal stress, after controlling for key socio-economic and demographic covariates. Results showed that both PHFI and HIV were independently associated with maternal stress. Yet, when both risk factors were present simultaneously there was a strong synergism on maternal stress. One possible explanation is that household food insecurity is likely to be a strong maternal stressor with important consequences for the mental health of pregnant women. This stress may be further exacerbated by HIV as HIV-positive women who may have the expectation of not being able to perform the tasks needed to secure food needed by the family. On the other hand food insecurity may amplify the stress of HIV by preventing women from taking proper care of their disease.

Previous studies have identified food insecurity as a mental health stressor. In rural Tanzania, Hadley and Patil [4] found that food insecurity increases the risk of mental health problems, such as anxiety and depression. Whitaker et al. [20] also found that there was a positive association between higher levels of food insecurity and higher levels of generalized maternal anxiety disorder. Food insecurity was found to increase distress in mothers after pregnancy [21]. Hromi-Fiedler et al. [22] found that low-income pregnant Latinas that were food insecure were more likely to be depressed.

As expected, in our study HIV-positive women were more likely to be psychologically and socio-economically vulnerable. HIV-positive mothers were more likely to perceive stress, were more likely to live in households that were persistently food insecure, to not have a spouse, to not have electricity at home, and to have lower levels of education. Specific causes for greater stress among HIV positive mothers are the possibility of transmitting the virus to their children and the potential inability to take care of the children after birth [23]. A recent study found that HIV-positive mothers were more likely to be depressed [24]. In a study conducted in New York City, Mellins et al. [25] found that a diagnosis of HIV is among the most traumatic stressful life events, and that parental stress and a greater number of household members were significantly associated with low adherence to treatment in HIV-positive mothers.

Our study design did not allow us to examine whether food insecurity lead to an increased risk of maternal HIV as we did not assess food insecurity prior to HIV diagnosis. On the other hand, however, our findings do suggest that an HIV diagnosis may have led to a worsening of the food insecurity situation in affected households. Whereas the levels of severe household food insecurity among HIV-positive mothers increased over time, HIV-negative mothers actually experienced a decrease. The increased divergence in between-group severe household food insecurity across time indeed suggests that HIV diagnosis may have led to the worsening of food insecurity in the households where HIV-positive women lived. Because this divergence became evident around 6-months post-partum it is important that future studies help understand if and how household food insecurity changes when there is a need to introduce complementary foods into the infant’s diet. Gibbs [26] argues that gender inequality makes households more food insecure due to the HIV and AIDS epidemic.

There were several limitations in this study. Although there was a significant relationship in being both HIV-positive and PHFI with perceived maternal stress, the sample size was relatively small. One reason is that PHFI was reported by only 25 (10.8%) of the women. This level of food insecurity is consistent with a relatively adequate level of maternal education (8^th^ grade on average) and socioeconomic status (e.g. 80% lived in households with electricity) in the study’s sample. Another reason is that HIV-positive women experienced greater attrition from the study, which is understandable considering that their greater socioeconomic and biological vulnerability makes them inherently less likely to continue in the study. Another limitation is that we didn’t have the statistical power needed to test different levels of persistence of HFI (e.g. being HFI three out of the four time points vs. throughout the study). This may have precluded us from better understanding how seasonal fluctuations in HFI may affect maternal stress levels. An additional limitation is that we did not have enough statistical power to formally test the full 2 × 2 interaction between HIV and HFI as only 7 women were both PHFI and HIV-negative. However the 3-level categorical variable modeled strongly suggests a high level of synergism between HIV and PHFI on maternal stress. Lastly, our analyses can’t determine whether HIV diagnosis led to PHFI, if PHFI led to high-risk behaviors that caused the HIV infection, or both. Indirect evidence from studies in other settings suggests that this relationship is indeed bidirectional. Weiser et al. [9] found that food insecurity and insufficiency can increase the risk of HIV infection and transmission among women. Transactional sex, intergenerational sex, and difficulties in negotiating condom use can result from stressors such as being a single mother [27]. In the other hand, findings do suggest that the risk of severe household food insecurity is likely to increase as a result of HIV diagnosis.

The majority of studies on HIV-positive mothers have been related to vertical transmission; thus, there is a dearth of research on stigma and coping mechanisms associated with both maternal HIV diagnosis [28-30] and household food insecurity [31]. This gap needs attention as maternal-child mental health has been identified as priority in Sub-Saharan Africa and globally [32,33]. The strong synergy between PHFI and being HIV-positive on maternal stress has important implications for women’s health and the development of their children. Studies have shown that exposure to maternal stress in early childhood has long lasting effects on the psychological health of the child [34-36]. Clearly, intervention studies are needed to better understand how to reduce stress among HIV-positive women living in households experiencing persistent food insecurity. If effective, these interventions are likely to translate into better maternal health and child development outcomes. In addition, addressing household food insecurity may also help buffer women against the negative influence of HIV on maternal stress.

## Conclusions

Findings from this study have important public policy implications. Most programs targeting HIV affected individuals or communities have strictly focused on HIV prevention, counseling and treatment without taking into account social inequality as the root of the epidemic and linked syndemics. Addressing maternal stress as a result of the interaction between food insecurity and HIV requires comprehensive multi-dimensional interventions that go beyond HIV prevention, counseling and treatment and include sustainable poverty reduction and community development, and education in addition to HIV treatment [6-8]. Our findings suggest that mental health should be an integral part of addressing maternal care in communities affected by the HIV-food insecurity syndemic. This recommendation has immediate implications for Ghana where the second HIV/AIDS National Strategic Framework implemented for the period of 2006–2010, did not fully address the need for understanding how to handle the risk of mental health problems, including mental stress, associated with an HIV diagnosis [13], and does not recognize that this risk becomes highly amplified in the presence of household food insecurity.

## Appendix

### Questions from the adapted USDA household food security survey module used in RIING study (adult/household items)

“We worried whether our food would run out before we could get more.” Was that often true, sometimes true, or never true for your household in the last month?

“The food that we had just didn’t last, and we couldn’t get more.” Was that often, sometimes, or never true for your household in the last month?

“We couldn’t get the kinds of foods that give us good health.” Was that often, sometimes, or never true for your household in the last month?^1^

In the last month, did (you/you or other adults in your household) ever reduce the amount of food you ate or skip meals because there wasn’t enough food?

In the last month, did you ever eat less because there wasn't enough food?

In the last month, were you ever hungry but didn't eat because there wasn’t enough food?

In the last month, did you lose weight because you didn't have enough food to eat?

In the last month, did you or other adults in your household ever not eat for a whole day because there wasn't enough food?

^1^This question was excluded because it did not fit well with the rest of scale according to Rasch analyses. Thus, the RIING study’s FI score was based on the 7 remaining items.

## Competing interests

None of the authors has any potential conflict of interest to report.

## Authors’ contributions

RPE, RM, GM, DS and AL designed study. JG, AHF and RPE conducted data analyses. JG and RPE wrote first manuscript draft. All authors participated in results interpretation and reviewed the manuscript. All authors read and approved the final manuscript.

## Pre-publication history

The pre-publication history for this paper can be accessed here:

http://www.biomedcentral.com/1471-2458/13/215/prepub
